# Vitamin B Supplementation for Diabetic Peripheral Neuropathy: A Review and Meta-Analysis

**DOI:** 10.3390/jcm15135156

**Published:** 2026-07-02

**Authors:** Ekaterina V. Mandra, Vladimir A. Parfenov, Victor A. Stupin, Ekaterina V. Silina

**Affiliations:** 1 Department of Nervous System Diseases, I.M. Sechenov First Moscow State Medical University (Sechenov University), 119991 Moscow, Russia; mandra_e_v@staff.sechenov.ru (E.V.M.); parfenov_v_a@staff.sechenov.ru (V.A.P.); 2 Department of Hospital Surgery, Pirogov Russian National Research Medical University, 117997 Moscow, Russia; stvictor@bk.ru

**Keywords:** diabetic peripheral neuropathy, vitamin B, vitamin B complex, diabetic neuropathies, neuropathy, diabetes, pain, nerve conduction, benfotiamine, methylcobalamin, meta-analysis

## Abstract

**Background/Objectives:** Diabetic peripheral neuropathy (DPN) is a common and disabling complication of diabetes. B vitamins play essential roles in neuronal metabolism, but evidence on their therapeutic efficacy remains inconsistent. The review and meta-analysis aimed to evaluate the effects of vitamin B supplementation on clinical and neurophysiological outcomes in DPN. **Methods:** We searched PubMed, Cochrane Database, and ClinicalTrials.gov, supplemented by reference screening of relevant systematic reviews. Randomized controlled trials (RCTs) comparing vitamin B (any form, dose, route) versus placebo or standard care in adults with DPN were included. Primary outcomes were pain intensity, neuropathy scores, and nerve conduction parameters. Risk of bias was assessed using RoB 2. Random-effects meta-analyses were performed where appropriate. **Results:** Thirteen RCTs (834 participants) were included. Vitamin B supplementation significantly improved Michigan Neuropathy Screening Instrument Questionnaire (MNSIQ: MD −1.44, 95% CI −2.48 to −0.39) and Examination (MNSIE: MD −0.39, 95% CI −0.66 to −0.12). However, the improvement in MNSIE did not reach the established minimal clinically important difference (MCID) of 0.5 points. Pain intensity on numeric/visual analogue scales showed no significant effect (MD −0.44, 95% CI −1.77 to 0.89; high heterogeneity, I^2^ = 84.5%), but disease-specific pain scales favoured vitamin B (MD −3.06, 95% CI −5.61 to −0.51). Sural nerve conduction velocity (MD 2.10 m/s, 95% CI 0.35 to 3.86) and amplitude (MD 0.88 µV, 95% CI 0.08 to 1.67) improved significantly. Peroneal and tibial nerve parameters showed no consistent benefit; tibial velocity favoured control (MD −1.23 m/s, 95% CI −2.37 to −0.09). Combination B-vitamin regimens appeared more effective than monotherapy. Formal subgroup analysis did not demonstrate a statistically significant difference between monotherapy and combination therapy for MNSIE, MNSIQ, or sural nerve NCV (interaction *p* > 0.05) but demonstrated one for peroneal nerve NCV (interaction *p* = 0.039), although a numerical trend towards greater benefit with combination regimens was observed. Risk of bias was low in six studies, some concerns in five, and high in two. **Conclusions:** Vitamin B supplementation may improve certain clinical neuropathy scores and sural nerve function in DPN, but effects on pain and motor nerve parameters are inconsistent. Combination therapy (multiple B vitamins) seems more promising than single agents. However, substantial heterogeneity and risk of bias preclude firm recommendations for routine use. Well-designed RCTs stratified by baseline vitamin status are needed.

## 1. Introduction

Diabetic peripheral neuropathy (DPN) is one of the most prevalent and debilitating complications of diabetes mellitus, affecting an estimated 25% to 50% of individuals with diabetes over their lifetime [1,2]. With global diabetes prevalence projected to exceed 578 million by 2030 and 700 million by 2045, the burden of DPN will proportionally increase [3,4]. This condition substantially diminishes quality of life, increases the risk of foot ulcers and lower-extremity amputations, and imposes a significant economic burden on healthcare systems worldwide [5,6,7,8].

The pathogenesis of DPN is complex and multifactorial, with chronic hyperglycemia triggering metabolic, vascular, and oxidative stress pathways that lead to peripheral nerve damage. These include enhanced polyol pathway flux, advanced glycation end-product formation, protein kinase C activation, mitochondrial dysfunction, and neuroinflammation [9,10,11,12,13]. Deficiencies of B vitamins have been consistently associated with diabetic neuropathy, and supplementation may serve as an effective therapeutic strategy [7,14,15,16,17].

B vitamins serve as critical cofactors in neuronal metabolism. Thiamine (B1) and its derivative benfotiamine play vital roles in glucose metabolism and block hyperglycemia-induced damage pathways [18]. Pyridoxine (B6) modulates neuronal excitability through neurotransmitter synthesis [19], and deficiency has been reported in 51.8% of diabetic neuropathy patients [20]. Cobalamin (B12) acts as an essential methyl donor in DNA metabolism and promotes myelin maintenance [21,22,23,24]. Folate (B9), together with B6 and B12, plays a crucial role in homocysteine metabolism, with deficiencies leading to hyperhomocysteinemia and increased oxidative stress [25,26]. Preclinical studies have confirmed that folic acid deficiency impairs nerve regeneration, while supplementation restores Schwann cell proliferation [27,28,29]. The C677T variant of the methylenetetrahydrofolate reductase gene is associated with greater DPN risk, and L-methylfolate has shown promise for symptomatic relief [30].

Patients with diabetes frequently exhibit B-vitamin deficiencies. Metformin, the first-line agent for type 2 diabetes, is consistently associated with reduced serum B12 levels through interference with calcium-dependent absorption of the intrinsic factor–vitamin B12 complex [31,32,33]. This drug–nutrient interaction is dose-dependent, with a 1 g/day increase in metformin dose associated with a 13% higher risk of B12 deficiency, and routine screening is recommended by the American Diabetes Association [34,35,36,37,38]. Metformin-induced B12 deficiency can induce or exacerbate peripheral neuropathy [39,40,41,42]. Gao et al. (2023) found that patients receiving ≥1500 mg/day metformin had significantly higher prevalence of borderline B12 deficiency (16.76% vs. 9.91%) [43]. Additionally, increased urinary excretion of thiamine and reduced plasma levels of pyridoxal-5′-phosphate have been observed in diabetic populations [44,45,46,47].

Given these mechanistic links, supplementation with B vitamins has been proposed as a therapeutic strategy for DPN. However, systematic reviews and meta-analyses have reached inconsistent conclusions. A 2008 Cochrane review concluded that insufficient evidence existed to determine whether vitamin B supplementation is beneficial or harmful for peripheral neuropathy [48]. More recent meta-analyses have provided conflicting findings: Huo et al. reported that vitamin and antioxidant supplements might improve nerve conduction velocity, though no treatment effect was observed for B12 specifically [49]. Farah and Yammine found improvements in pain symptoms and electrophysiological parameters [50]. Stein et al. demonstrated associations between B-vitamin status and neuropathy but emphasized the need for larger trials [51]. Pratama et al. (2022) concluded that B12 supplementation for metformin-treated patients improves neuropathy symptoms [52,53,54]. The heterogeneity of these findings reflects variations in study populations, intervention protocols, and outcome measures. Additionally, the presence of underlying vitamin deficiencies—which varies across populations—could influence treatment response [55].

Existing systematic reviews and meta-analyses on vitamin B in DPN have several limitations that our updated review directly addresses: (1) Ang et al. (2008) [48] predates the majority of available RCTs and did not include any of the trials published after 2008 (10 of our 13 included studies); (2) Farah & Yammine (2022) [50] focused on four studies and did not provide nerve-specific electrophysiological analyses by nerve type; (3) Karedath et al. (2022) [55] restricted inclusion to vitamin B12 monotherapy and excluded combination regimens and other B vitamins; (4) Huo et al. (2023) [49] grouped vitamin B with antioxidants and did not assess clinical neuropathy scores (MNSI). The present review is the first to: (i) synthesize all forms of vitamin B (B1, B6, B9, B12, combinations) across the full spectrum of DPN outcomes (pain, clinical scores, and nerve-specific electrophysiological parameters); (ii) include the most recently published RCTs (through March 2026); (iii) perform formal subgroup analyses comparing monotherapy versus combination therapy.

Therefore, the present review and meta-analysis aims to: (1) evaluate the impact of vitamin B supplementation on the treatment of DPN; (2) explore potential effect modifiers (vitamin subtype, combination vs. monotherapy). Planned subgroup analyses to identify additional effect modifiers (dose, duration, route, baseline vitamin status) were pre-specified but could not be performed due to an insufficient number of studies per subgroup.

By comprehensively synthesizing randomized controlled trials and exploring potential sources of heterogeneity, this review seeks to provide clearer guidance for clinical practice and identify priorities for future research in this clinically important area.

## 2. Materials and Methods

The systematic review was reported in accordance with the Preferred Reporting Items for Systematic Reviews and Meta-Analyses (PRISMA) 2020 statement [56], which helps ensure transparency and reproducibility of the results.

### 2.1. Search Strategy

A comprehensive literature search was conducted in the electronic databases PubMed, Cochrane Database and ClinicalTrials.gov (accessed on 14 March 2026). The search strategy combined terms for diabetic neuropathy and vitamin B, including specific vitamin names and derivatives: (diabetic AND neuropathy) AND (“vitamin B” OR “B vitamins” OR “thiamine” OR “vitamin B1” OR “benfotiamine” OR “vitamin B6” OR “pyridoxine” OR “vitamin B12” OR “cobalamin” OR “methylcobalamin” OR “folic acid” OR “folate” OR “vitamin B9”). In addition, reference lists of relevant systematic reviews and meta-analyses were screened to identify further eligible studies. The following reviews were used as source publications: Stein et al. (2021) [51]; Huo et al. (2023) [49]; Ang et al. (2008) [48]; Karedath et al. (2022) [55]; Farah & Yammine (2022) [50]; Khalil et al. (2021) [57]; Muhamad et al. (2023) [58]; Sawangjit et al. (2020) [59].

### 2.2. Eligibility Criteria

Studies were included if they met the following PICOS criteria:

Population

(a) Adults (≥18 years) with a diagnosis of diabetic peripheral neuropathy (type 1 or type 2 diabetes); (b) diagnosis confirmed by at least one of the following: validated clinical scales (e.g., Neuropathy Disability Score, Michigan Neuropathy Screening Instrument, Total Symptom Score), nerve conduction studies, or explicit diagnostic criteria described by the authors; (c) no restrictions on sex, ethnicity, or disease duration.

Intervention

(a) Administration of any vitamin B (for example, thiamine/B1, benfotiamine, pyridoxine/B6, cobalamin/B12, folate/B9, or combinations) in any dose, route (oral, intramuscular, intravenous), and duration; (b) allowed as monotherapy or added to standard care, provided the control group received the same standard care.

Comparator

This was placebo, no treatment (standard care only), or active control where the effect of vitamin B could be isolated (e.g., vitamin B + standard care vs. placebo + standard care).

Outcomes

Studies had to report at least one of the following outcomes with extractable quantitative data: (a) pain intensity (visual analogue scale, numerical rating scale, or other validated scale); (b) neuropathy clinical scores (e.g., NDS, NSS, TSS, MNSI); (c) nerve conduction parameters (motor or sensory nerve conduction velocity, amplitude, latency) for specified nerves; (d) secondary outcomes: quality of life, adverse events, biochemical markers (e.g., vitamin levels, homocysteine).

Study design

Only randomized controlled trials (parallel-group, crossover with adequate washout, or cluster-randomized) were eligible. No language restrictions were applied.

### 2.3. Exclusion Criteria

Studies were excluded if they: (a) were non-randomized (observational studies, case series, case reports, reviews, meta-analyses, commentaries, letters, protocols); (b) included patients with other types of neuropathy (e.g., alcoholic, hereditary, toxic) or mixed populations where data for DPN could not be extracted separately; (c) used vitamin B as a minor add-on component within a complex multi-agent intervention primarily designed to evaluate another therapeutic class, where B vitamins played a negligible or auxiliary role; (d) lacked an adequate control group (no placebo or standard care alone); (e) did not provide sufficient numerical data for meta-analysis (e.g., only qualitative statements or graphs without numbers, and authors did not respond to requests); (f) were duplicate publications (only the most complete report was included); (g) were animal or in vitro studies; (h) had no full text available after reasonable retrieval attempts through institutional access, publisher pages, DOI/PMID searches, and general web searches.

### 2.4. Data Extraction

A standardized data extraction form was used to collect the following information from each included study: (a) study characteristics: first author, year, country, study design, sample size, and duration of follow-up; (b) participant characteristics: age, sex, diabetes type, diabetes duration, baseline vitamin status, and HbA1c; (c) intervention details: type of vitamin B, dose, route, frequency, and duration; (d) comparator details: type of control (placebo, standard, etc.); (e) outcomes: means and standard deviations (or change scores) for continuous outcomes, number of events for dichotomous outcomes, and any other relevant data; (f) funding source and conflicts of interest. Data extraction was performed independently by at least two authors. Discrepancies were resolved by consensus discussion or, when consensus could not be reached, by consultation with a third senior reviewer.

### 2.5. Study Selection Process

**Figure 1:**
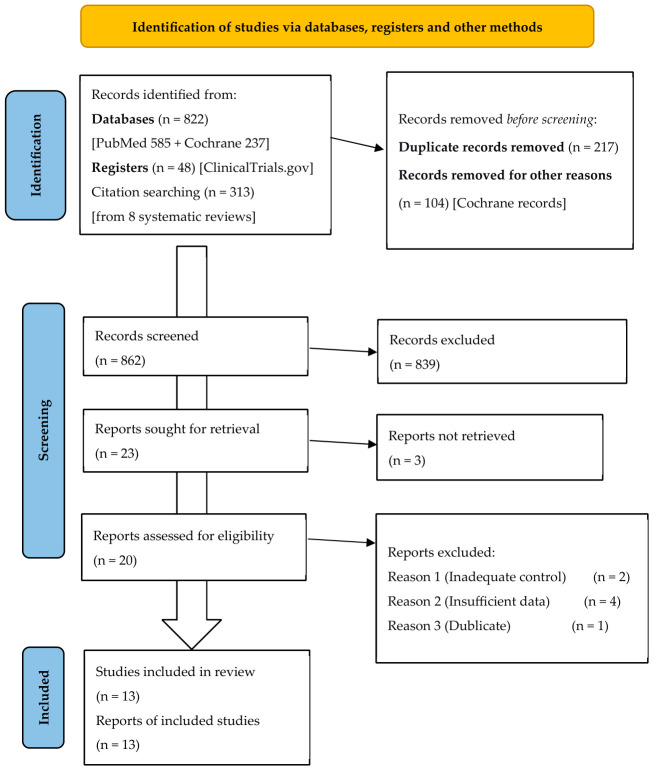
PRISMA flowchart of study selection strategy.

Three reviewers independently screened titles and abstracts of all retrieved records. Full texts of potentially relevant articles were obtained and assessed against the eligibility criteria. Disagreements were resolved through discussion or consultation with the fourth reviewer. The selection process was documented and is presented in the PRISMA flow diagram (Figure 1).

### 2.6. Risk-of-Bias Assessment

The risk of bias in included randomized control trials was assessed using the Cochrane Risk of Bias 2 (RoB 2) tool [60]. Each study was evaluated across five domains: randomization process, deviations from intended interventions, missing outcome data, measurement of the outcome, and selection of the reported result. Judgments were categorized as “low risk,” “some concern,” or “high risk.” Two reviewers independently performed the assessments, and disagreements were resolved by discussion.

### 2.7. Data Synthesis and Statistical Analysis

For continuous outcomes, mean differences (MDs) or standardized mean differences (SMDs) with 95% confidence intervals (CIs) were calculated. The random-effects model was the pre-specified primary analytical approach for all outcomes, regardless of heterogeneity level. Fixed-effect estimates are reported in supplementary analyses for transparency only and are clearly labelled as secondary. Heterogeneity was assessed using the I^2^ statistic and the Chi^2^ test (*p* < 0.05 considered significant). For all studies, a random-effects model was used. Sensitivity analyses were performed by sequentially omitting each study to examine its influence on the pooled estimate.

Planned subgroup analyses (if data allowed) included: (a) monotherapy vs. combination therapy; (b) type of vitamin B (B1, B6, B9, B12, combinations); (c) dose (low vs. high); (d) duration of supplementation (≤3 months vs. >3 months); (e) route of administration (oral vs. parenteral); (f) study quality (low vs. high risk of bias). Of these, only the monotherapy vs. combination analysis (subgroup a) had sufficient data (≥2 studies per subgroup) for most outcomes. The remaining subgroup analyses could not be performed due to insufficient studies per subgroup.

All statistical analyses were performed using Python (version 3.11; Python Software Foundation (Wilmington, DE, USA)), with the following packages: pandas (version 2.0), numpy (version 1.24), scipy (version 1.11), statsmodels (version 0.14), and plotly (version 5.18). The analysis code was created in JupyterLab (version 4.6.0).

Publication bias was assessed for outcomes with ≥5 studies using funnel plots and Egger’s test [61]. *p* < 0.05 was considered to be statistically significant. For outcomes with fewer than 5 studies, formal testing was not performed due to insufficient statistical power; funnel plots are provided for descriptive purposes only with appropriate caveats.

## 3. Results

### 3.1. Study Selection

The literature search yielded 870 records in total: 822 from electronic databases (PubMed: 585, Cochrane: 237) and 48 from ClinicalTrials.gov. Based on a preliminary assessment of Cochrane records, 104 were considered ineligible (Supplementary Material S1) and were excluded at this stage, leaving 658 records from databases for further processing.

Reference screening of eight systematic reviews initially identified 390 cited records. During preliminary review, 77 records were classified as clearly off-topic for the present review question and were not carried forward. Therefore, 313 candidate records from review reference lists were retained (Supplementary Material S1). After removing duplicates within these reviews (n = 53), 260 unique records were added.

The database records (n = 658) and review-derived records (n = 260) were combined, yielding 918 records. After removing duplicates between these two sources (n = 56), a total of 862 unique records were screened at the title and abstract level.

During title/abstract screening, 839 records were excluded as they did not meet the inclusion criteria (Supplementary Material S1). The remaining 23 reports were sought for full-text retrieval. Three full texts could not be obtained, leaving 20 reports for detailed eligibility assessment.

After full-text evaluation, seven reports were excluded for the following reasons: inadequate control group (n = 2), insufficient data for meta-analysis (datasets without statistics (n = 4)) and same-cohort analysis (duplicate (n = 1)).

Thus, 13 randomized controlled trials met all inclusion criteria and were included in the systematic review. The PRISMA flow diagram (Figure 1) summarizes the selection process.

### 3.2. Study Characteristics

The 13 included trials [62,63,64,65,66,67,68,69,70,71,72,73,74] comprised a total of 834 participants (414 in intervention groups, 420 in control groups). Sample sizes ranged from 14 to 214 participants. Studies were conducted across multiple countries, including Greece, Indonesia, the United States, Iran, Germany, Malaysia, and Norway. The dates of publication ranged from 1981 to 2026.

The included trials were substantially heterogeneous across several dimensions: (1) interventions ranged from B12 monotherapy (i.v. or oral) to complex multi-micronutrient formulations; (2) treatment duration ranged from 0.28 months (five i.v. injections over 9 days) to 24 months; (3) comparators included placebo, no treatment, and active comparators (amitriptyline, nortriptyline); (4) three studies enrolled exclusively or predominantly type 1 diabetes patients (Fraser 2012) [71], others type 2, and several mixed. Two studies (Didangelos 2020 and Farvid 2011) [62,66] evaluated interventions in which B vitamins were co-administered with non-B potentially neuroactive agents, including superoxide dismutase, alpha-lipoic acid, acetyl-L-carnitine, minerals, and antioxidant vitamins. Therefore, the specific contribution of B vitamins cannot be isolated in these studies. Fonseca 2013 [65] evaluated a B-vitamin combination (L-methylfolate, methylcobalamin, and pyridoxal-5′-phosphate) and should be interpreted as a multi-B-vitamin regimen rather than as a non-B co-intervention study.

The interventions varied across trials, including:

Vitamin B12 (cobalamin/methylcobalamin): administered orally (0.5–1 mg/day) or intravenously (0.5 mg).

Thiamine/Benfotiamine (vitamin B1): oral doses ranging from 120 to 600 mg/day.

Pyridoxine (vitamin B6): oral doses of 150 mg/day.

Folic acid (vitamin B9): 1 mg/day.

Combination therapies: various B-vitamin combinations.

Treatment duration ranged from 0.28 months (approximately 1 week) to 24 months. Control groups received placebo, no treatment, or active comparators (including nortriptyline, acetyl-L-carnitine, and various other agents). Baseline characteristics of included studies are presented in Table 1.

### 3.3. Risk-of-Bias Assessment Results

**Figure 2:**
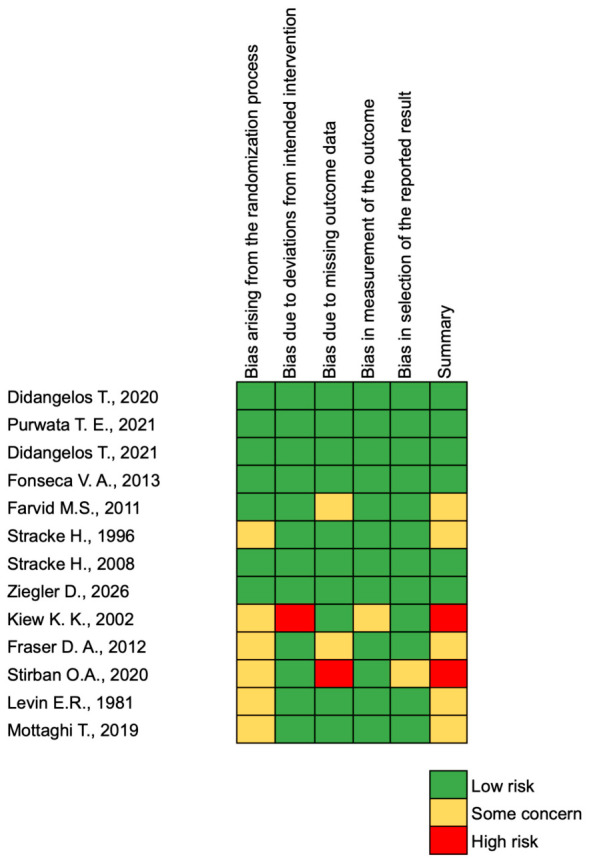
Risk-of-bias assessment of randomized controlled trials [62,63,64,65,66,67,68,69,70,71,72,73,74].

**Figure 3:**
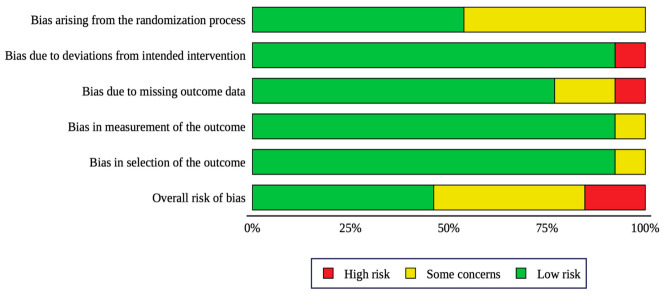
Risk-of-bias assessment graph of randomized controlled trials.

Risk of bias was assessed using the Cochrane Risk of Bias 2 (RoB 2) tool (Figure 2 and Figure 3). Of the 13 included RCTs, 6 studies (46.1%) were assessed as having low risk of bias overall; 5 studies (38.5%) were assessed as having some concerns; and 2 studies (15.4%) were assessed as having high risk of bias. The primary sources of bias concerns were related to the randomization process and measurement of the outcome.

### 3.4. Pain Intensity

**Figure 4:**
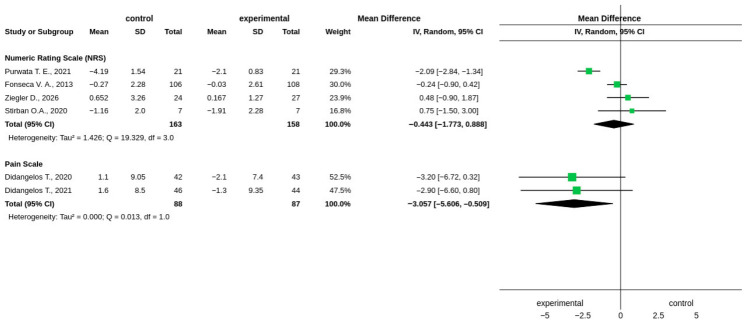
Intervention effect of B-vitamin supplements compared to control on pain in DPN. The green square represents the effect estimate for each individual study, with the square size reflecting the weight of that study in the meta-analysis; the horizontal line indicates the 95% confidence interval; the black diamond shows the pooled effect estimate from the random-effects model [62,63,64,65,69,72].

Four studies (n = 321 participants) reported pain intensity using validated scales (NRS/VAS). Due to substantial heterogeneity (Q = 19.329, df = 3, I^2^ = 84.5%), a random-effects model was employed (Figure 4).

Due to extreme statistical heterogeneity (I^2^ = 84.5%, τ^2^ = 1.426), the pooled random-effects estimate (MD −0.443; 95% CI −1.773, 0.888) should not be interpreted as a reliable summary of treatment effect, following Cochrane guidance against pooling when I^2^ > 75% and heterogeneity is unexplained. A narrative synthesis is more appropriate: three of four studies showed effects near zero (Didangelos 2020, Didangelos 2021, Fonseca 2013) [62,64,65], while one extreme outlier (Purwata 2021) [63] showed a large effect (MD −2.09) with short treatment duration (9 days) and amitriptyline in both arms.

The pooled analysis showed no statistically significant difference between vitamin B supplementation and control groups (random-effects MD = −0.443, 95% CI: [−1.773, 0.888]). The fixed-effect model suggested a significant difference (MD = −0.806, 95% CI: [−1.262, −0.351]), but this result is unreliable given the high heterogeneity.

Visual inspection of the forest plot revealed a notable outlier (Purwata et al., 2021) [63] showing a strong effect favouring the experimental group, while the remaining studies showed effects clustering near zero. Of note, the study employed tricyclic antidepressant (amitriptyline) in both arms, which may influence the interpretability of the effect size.

Two studies (Didangelos et al., 2020 [62]; Didangelos et al., 2021 [64]; n = 175 participants) reported pain outcomes using a validated pain scale (PS), which correspond to the Pain Detect Questionnaire. Heterogeneity was low (Q = 0.01, df = 1, I^2^ = 0%). The pooled analysis demonstrated a statistically significant reduction in pain scores favouring vitamin B supplementation (fixed-effect MD = −3.06, 95% CI: [−5.61, −0.51]). However, this result should be interpreted with extreme caution: both studies originate from the same research group and the same institution, and use the same intervention protocol. The I^2^ = 0% is an expected mathematical consequence of pooling only two studies and cannot be interpreted as evidence of cross-study consistency. This finding requires independent replication.

These results suggest that while the effect on generic NRS pain intensity was not statistically significant across all studies, the more disease-specific pain scales (PSs) showed a consistent and significant benefit of vitamin B therapy. However, the limited number of studies using PSs warrants cautious interpretation.

### 3.5. Neuropathic Symptoms

Michigan Neuropathy Screening Instrument Questionnaire (MNSIQ): Four studies (n = 233 participants) reported MNSIQ scores. Heterogeneity was substantial (Q = 15.599, df = 3, I^2^ = 80.1%). The random-effects pooled estimate demonstrated a statistically significant improvement favouring vitamin B supplementation (MD = −1.435, 95% CI: [−2.476, −0.394]).

**Figure 5:**
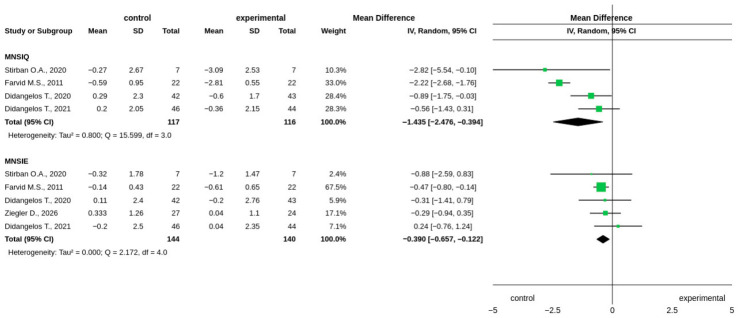
Intervention effect of B-vitamin supplements compared to control on neuropathic symptoms in DPN. The green square represents the effect estimate for each individual study, with the square size reflecting the weight of that study in the meta-analysis; the horizontal line indicates the 95% confidence interval; the black diamond shows the pooled effect estimate from the random-effects model [62,64,66,69,72].

Michigan Neuropathy Screening Instrument Examination (MNSIE): Five studies (n = 284 participants) reported MNSIE scores. Heterogeneity was low (Q = 2.177, df = 4, I^2^ = 0%). The pooled analysis showed a statistically significant improvement favouring vitamin B supplementation (fixed-effects MD = −0.389, 95% CI: [−0.655, −0.123]) (Figure 5).

### 3.6. Nerve Conduction Velocity

**Figure 6:**
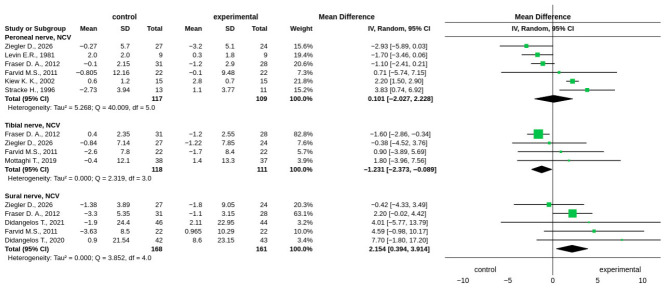
Intervention effect of B-vitamin supplements compared to control on NCV in DPN. The green square represents the effect estimate for each individual study, with the square size reflecting the weight of that study in the meta-analysis; the horizontal line indicates the 95% confidence interval; the black diamond shows the pooled effect estimate from the random-effects model [62,64,66,67,69,70,71,73,74].

Peroneal Nerve NCV: Six studies (n = 226 participants) reported peroneal nerve conduction velocity. Heterogeneity was extremely high (Q = 40.394, df = 5, I^2^ = 87.6%). The random-effects pooled estimate showed no statistically significant difference between groups (MD = 0.093, 95% CI: [−2.037, 2.223]). The high heterogeneity reflects divergent study results, with some trials favouring treatment and others favouring the control (Figure 6).

Tibial Nerve NCV: Four studies (n = 229 participants) reported tibial nerve conduction velocity. Heterogeneity was low (Q = 2.330, df = 3, I^2^ = 0%). The pooled analysis demonstrated a statistically significant difference favouring the control group (MD = −1.227, 95% CI: [−2.366, −0.087]).

Sural Nerve NCV: Five studies (n = 329 participants) reported sural nerve conduction velocity. Heterogeneity was moderate (Q = 4.024, df = 4, I^2^ = 0.6%). The pooled analysis showed a statistically significant improvement favouring vitamin B supplementation (MD = 2.102, 95% CI: [0.345, 3.860]).

### 3.7. Nerve Amplitude

**Figure 7:**
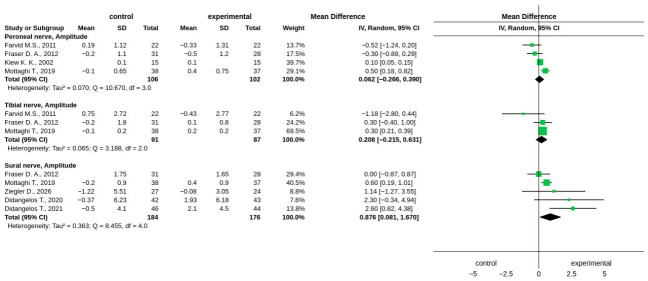
Intervention effect of B-vitamin supplements compared to control on nerve amplitude in DPN. The green square represents the effect estimate for each individual study, with the square size reflecting the weight of that study in the meta-analysis; the horizontal line indicates the 95% confidence interval; the black diamond shows the pooled effect estimate from the random-effects model [62,64,66,69,70,71,74].

Peroneal Nerve Amplitude: Four studies (n = 208 participants) reported peroneal nerve amplitude. Heterogeneity was moderate (Q = 10.670, df = 3, I^2^ = 71.9%). The random-effects pooled estimate showed no statistically significant difference between groups (MD = 0.062, 95% CI: [−0.266, 0.390]) (Figure 7).

Tibial Nerve Amplitude: Three studies (n = 178 participants) reported tibial nerve amplitude. Heterogeneity was low (Q = 3.188, df = 2, I^2^ = 37.3%). The pooled analysis showed no statistically significant difference between groups (MD = 0.208, 95% CI: [−0.215, 0.631]).

Sural Nerve Amplitude: Five studies (n = 360 participants) reported sural nerve amplitude. Heterogeneity was moderate (Q = 8.460, df = 4, I^2^ = 52.7%). The random-effects pooled estimate demonstrated a statistically significant improvement favouring vitamin B supplementation (MD = 0.876, 95% CI: [0.083, 1.669]).

### 3.8. Publication Bias

Publication bias was assessed for all outcomes with ≥5 studies. For outcomes with four studies, Egger’s test is reported with the explicit caveat that statistical power is insufficient. Results are presented in (Table 2).

### 3.9. Subgroup Analysis: Monotherapy vs. Combination Therapy

Studies were classified as monotherapy (n = 9 studies: Purwata T. E., 2021 [63]; Didangelos T., 2021 [64]; Stracke H., 2008 [68]; Ziegler D., 2026 [69]; Kiew K. K., 2002 [70]; Fraser D. A., 2012 [71]; Stirban O.A., 2020 [72]; Levin E.R., 1981 [73]; Mottaghi T., 2019 [74]) or combination therapy (n = 4 studies: Didangelos T., 2020 [62]; Fonseca V. A., 2013 [65]; Farvid M.S., 2011 [66]; Stracke H., 1996 [67]). The subgroup analysis was performed for outcomes with ≥2 studies per subgroup.

**Figure 8:**
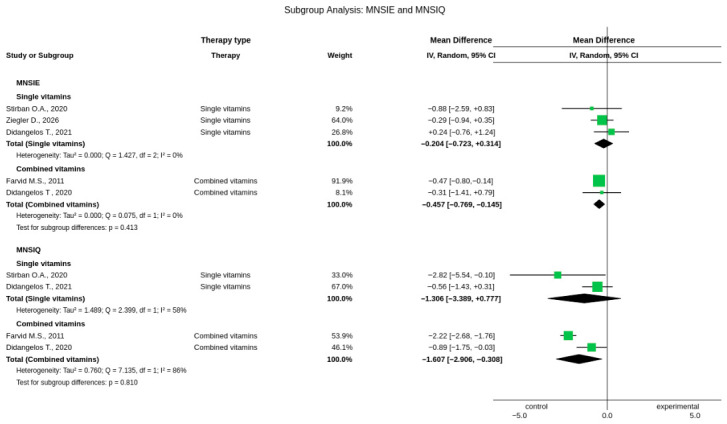
Intervention effect of combined vitamin supplements compared to single vitamins on neuropathic symptoms in DPN. The green square represents the effect estimate for each individual study, with the square size reflecting the weight of that study in the meta-analysis; the horizontal line indicates the 95% confidence interval; the black diamond shows the pooled effect estimate from the random-effects model [62,64,66,69,72].

**Figure 9:**
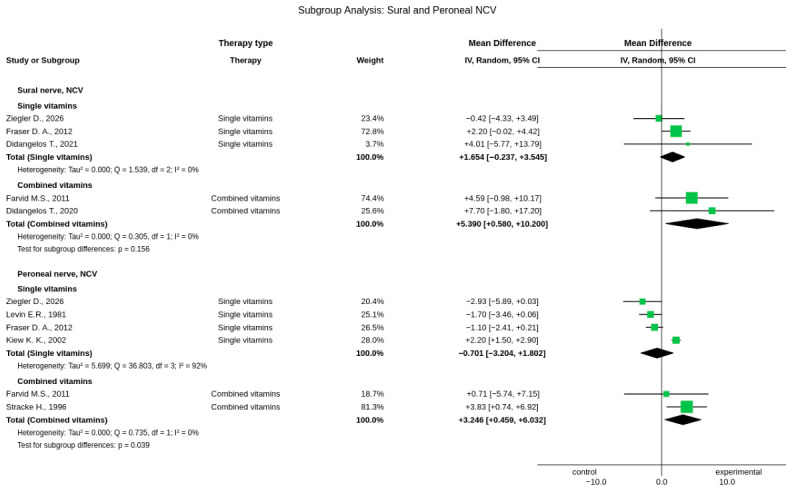
Intervention effect of combined vitamin supplements compared to single vitamin on NCV in DPN. The green square represents the effect estimate for each individual study, with the square size reflecting the weight of that study in the meta-analysis; the horizontal line indicates the 95% confidence interval; the black diamond shows the pooled effect estimate from the random-effects model [62,64,66,67,69,70,71,73].

There was a consistent numerical trend towards larger effects in combination therapy subgroups. Most of the interaction tests did not reach statistical significance (Figure 8 and Figure 9), except peroneal nerve NCV (Figure 9). Therefore, formal subgroup analysis does not confirm statistically significant superiority of combination over monotherapy for any outcome. These findings are hypothesis-generating and require dedicated head-to-head RCTs.

### 3.10. Leave-One-Out Sensitivity Analysis

Leave-one-out (LOO) sensitivity analysis was performed for all outcomes with ≥3 studies. All LOO estimates for MNSIE remain significant (range: −0.33 to −0.44). The result is robust to the exclusion of any single study. For MNSIQ, LOO estimates range from −0.96 to −2.07. Significance is maintained in 3/4 leave-one-out scenarios, which is moderately robust. Excluding Purwata 2021 [63] from consideration of NRS pain estimates leads to that effect disappearing entirely, confirming this single study drives the entire observed effect and heterogeneity. For Sural NCV, all LOO estimates remain significant (range: MD 1.28 to 2.84). For Sural Amplitude, LOO estimates range 0.49–1.15. A total of 4/5 scenarios remain significant. Peroneal NCV LOO estimates range −0.87 to 1.12, all non-significant; no single study drives this result; and there is no consistent direction.

### 3.11. Adverse Events

Four of the 13 included trials reported adverse events. No serious adverse events attributable to vitamin B supplementation were reported in any trial. The available adverse event data are insufficient for a formal safety meta-analysis. Based on available reports, oral and intravenous B-vitamin supplementation was generally well-tolerated in the included populations.

## 4. Discussion

This systematic review and meta-analysis of 13 randomized controlled trials provides an updated evaluation of the effects of vitamin B supplementation (alone or in combination) on clinical and neurophysiological outcomes in patients with DPN. Our findings reveal a complex and inconsistent picture: while certain outcomes showed statistically significant improvements, others did not, and substantial heterogeneity was observed across several analyses, underscoring the influence of individual study characteristics and the need for cautious interpretation.

Both MNSIQ and MNSIE showed statistically significant benefits with vitamin B supplementation. For MNSIE, heterogeneity was zero (τ^2^ = 0.0000), and the pooled estimate demonstrated a modest but consistent improvement (mean difference −0.390; 95% CI [−0.657, −0.122]). This finding is in line with earlier reports that combined B-vitamin preparations may improve neuropathic signs and symptoms [51]. Notably, the absence of heterogeneity for MNSIE suggests that the treatment effect is robust across different study designs and populations. However, the observed improvement in MNSIE did not reach the established minimal clinically important difference (MCID) of 0.5 points, indicating that while statistically significant, the magnitude of change may be below the threshold that patients perceive as clinically meaningful. For the MNSIQ, a validated MCID has not been established, limiting the interpretability of the observed change.

In contrast, the effect on pain intensity (NRS) did not reach statistical significance in the random-effects model (MD −0.437; 95% CI [−1.763, 0.889]), with high heterogeneity (τ^2^ = 1.426). This result mirrors the conclusions of the Cochrane review by Ang et al. [48], which found insufficient evidence to determine whether vitamin B is beneficial for neuropathic pain [75,76]. The heterogeneity was largely driven by one study (Purwata et al., 2021 [63]), which showed a strong effect favouring vitamin B supplementation. When this outlier was conceptually removed, the overall estimate became neutral, indicating that the negative result for pain may not be robust and could be influenced by these trials. For comparison, a recent systematic review on folate (vitamin B9) by Maues et al. (2025) demonstrated that folate supplementation reduced pain by up to 3 points on the VAS and improved Neuropathy Total Symptom Score-6 (NTSS-6) by 0.9–1.5 points, with effect sizes ranging from medium to large [77].

Neurophysiological outcomes diverged markedly across different nerves. For the sural nerve, both NCV and amplitude showed significant improvements with vitamin B supplementation (NCV: MD 2.102 m/s, 95% CI [0.345, 3.860]; amplitude: MD 0.876 µV, 95% CI [0.083, 1.669]), with low to moderate heterogeneity. All included studies pointed in the same direction (favouring treatment), making this the most consistent finding of our meta-analysis. This supports the hypothesis that B vitamins, particularly when given as part of a combination, may have a neuroprotective effect on sensory fibres, as previously suggested by some authors [47,78,79,80]. In line with this, a meta-analysis by Deng et al. (2025) investigating dapagliflozin combined with methylcobalamin reported significant improvements in common peroneal and sural nerve conduction velocities [81].

In contrast, peroneal nerve parameters did not show any significant pooled effect (NCV: MD 0.093 m/s, 95% CI −2.037 to 2.233; amplitude: MD 0.062 µV, 95% CI [−0.266, 0.390]), and heterogeneity was extreme (τ^2^ = 5.30 for velocity). Studies were split: some favoured treatment (benfotiamine–B6–B12 combination [67]; sulbutiamine [70]), while others favoured the control (benfotiamine [69,71]). Also, the meta-analysis and trial sequential analysis by Yu et al. (2026) found that vitamin B12 acupoint injection improved clinical effectiveness proportion by 28% compared to other administration routes, with significant improvements in peroneal and median nerve conduction velocities [82]. This contradictory evidence precludes any firm conclusion.

For the tibial nerve, the pooled effect favoured the control group (MD −1.227 m/s; 95% CI [−2.366, −0.087]) with no heterogeneity. This unexpected result was driven by a single large, precise study (Fraser 2012) [71] that showed a benefit of the control over vitamin B (benfotiamine). This should not be interpreted as evidence of harm from vitamin B but rather reflects the dominance of one study with an atypical direction of effect. Similar discrepancies have been noted in previous meta-analyses where the inclusion of individual high-precision trials altered the overall conclusion [51].

The differential effect of B-vitamin supplementation across nerve types warrants deeper consideration. Sural nerve parameters improved significantly in our meta-analysis, whereas peroneal nerve parameters showed no consistent benefit and tibial NCV unexpectedly favoured the control group. The sural nerve is a purely sensory nerve, while the peroneal and tibial nerves carry both motor and sensory fibres. In early and moderate DPN, sensory neuropathy typically predominates, reflecting the preferential vulnerability of sensory axons to hyperglycaemia-induced metabolic toxicity and the length-dependent “dying-back” axonopathy [3,9]. The biochemical targets of B vitamins—transketolase activation (thiamine), neurotransmitter synthesis (pyridoxine), and methylation cycle support (cobalamin, folate)—may preferentially support metabolic repair of sensory axons. Moreover, motor fibres are larger and more heavily myelinated; while methylcobalamin promotes myelin synthesis [21,22,23,24], the structural complexity of motor axons may require longer treatment durations or higher doses than those used in the included trials. The unexpected tibial NCV result (favouring the control) is most likely explained by the dominance of a single large study (Fraser 2012, n = 59, 24-month benfotiamine monotherapy in type 1 diabetes) [71]. This atypical result may reflect type 1 diabetes-specific pathophysiology, the specific form of B1 used, or chance. Given zero heterogeneity (τ^2^ = 0), this single study completely determines the pooled estimate, and the result should be interpreted cautiously as reflecting benfotiamine monotherapy in type 1 diabetes rather than B vitamins in DPN generally.

For comparison, a recent Cochrane review (Baicus et al., 2024) found little or no effect of alpha-lipoic acid on neuropathy symptoms measured by the Total Symptom Score (mean difference −0.16, 95% CI −0.83 to 0.51; MCID 0.97) and on impairment measured by the Neuropathy Impairment Score—Lower Limbs (mean difference −1.02, 95% CI −2.93 to 0.89; MCID 2) [83]. This contrast underscores that, unlike general antioxidants, B vitamins may exert more targeted effects on neuronal metabolism, although our own results remain heterogeneous and inconclusive.

The heterogeneity of our results allowed us to qualitatively assess the role of individual B vitamins. Vitamin B12 (methylcobalamin) as monotherapy [63,64] did not improve sural nerve conduction or pain; however, when combined with folate and pyridoxine [65], it contributed to improvement in neuropathy symptom scores and sural nerve parameters. Benfotiamine (a lipid-soluble B1 derivative) showed mixed results: Stracke 2008 [68] and Ziegler 2026 [69] did not demonstrate significant benefits on peroneal or tibial nerves, whereas [67] (benfotiamine combined with B6 and B12) showed some positive trends. Sulbutiamine (another B1 derivative) improved peroneal nerve amplitude and velocity [70], but this study had a high risk of bias (open-label, no blinding). Preclinical evidence suggests that thiamine and its derivative benfotiamine may protect against axonal degeneration through multiple mechanisms, including transketolase activation and reduction of advanced glycation end-products [84,85].

Pyridoxine alone [73] showed no significant effect on motor nerve conduction. Folic acid [74] improved several sensory and motor parameters of the tibial and peroneal nerves, but this effect was not replicated in study [66] where folic acid was given as part of a multivitamin mixture. Thus, the efficacy appears to depend not only on the specific vitamin but also on its formulation, dose, combination with other agents, and possibly the baseline nutritional status of patients.

Our results are partially consistent with earlier systematic reviews. Ang et al. [48] concluded that evidence was insufficient to determine whether vitamin B is beneficial or harmful for peripheral neuropathy. Huo et al. [49] found that vitamin and antioxidant supplements might improve nerve conduction velocity, but no treatment effect was observed for vitamin B12 specifically. Karedath et al. [55] reported that vitamin B12 can improve neuropathic symptoms and reduce pain, a finding that aligns with our results for MNSIQ and MNSIE but not for pain. Sawangjit et al. [59] concluded that mecobalamin (an active form of B12) in combination with other treatments may be effective for diabetic neuropathy, but the evidence was of low quality. Stein et al. [51] demonstrated associations between low B12 status and neuropathy, supporting biological plausibility. Farah and Yammine [50] reported improvements in pain and dysesthesia with vitamin B, which contrasts with our neutral pain result but agrees with our clinical score findings. The discrepancies may be explained by differences in included studies, outcome definitions, and statistical models.

While methylcobalamin alone [64] or benfotiamine alone [68,69] generally failed to demonstrate robust improvements in nerve conduction, combinations of benfotiamine with vitamins B6 and B12 [67] or L-methylfolate with methylcobalamin and pyridoxal-5′-phosphate [65] yielded significant benefits on sural nerve parameters and clinical scores. This observation aligns with the concept of metabolic synergy: thiamine blocks hyperglycemia-induced damage pathways, pyridoxine modulates neurotransmitter synthesis, and cobalamin promotes myelin repair. The negative trial by Fraser et al. [71] using benfotiamine monotherapy in type 1 diabetes further supports the hypothesis that single-agent B1 may be insufficient to counteract the multifactorial pathophysiology of DPN. A systematic review protocol by Lekhanya and Mokgalaboni (2022) on vitamin B12 complex and alpha-lipoic acid also emphasized that combination regimens yield more consistent neurophysiological improvements than monotherapy [86,87,88]. Future studies should prioritize testing standardized multi-B-vitamin combinations rather than individual vitamins.

A notable limitation of the evidence base is that two of the 13 included studies (Didangelos 2020 and Farvid 2011) [62,66] evaluated multi-component interventions that included non-B active or potentially neuroactive agents beyond B vitamins, including superoxide dismutase, alpha-lipoic acid, acetyl-L-carnitine, zinc, magnesium, and antioxidant vitamins C and E. While these studies compared the complete vitamin-containing intervention with placebo, it is impossible to definitively attribute the observed effects to B vitamins specifically. Fonseca 2013 [65] evaluated a combination of three B-vitamin forms (L-methylfolate, methylcobalamin, and pyridoxal-5′-phosphate) and is therefore a multi-B-vitamin regimen rather than a non-B co-intervention study. The pooled estimates influenced by Didangelos 2020 and Farvid 2011 should therefore be interpreted with this caveat [62,66].

An important factor that may modify the response to vitamin B supplementation is the patient’s baseline vitamin status. In the present meta-analysis, only two of the 13 included trials reported baseline serum B12 status, and no trial stratified randomisation or reported treatment effects according to baseline deficiency status [62,64]. Recent reviews have emphasized that treatment response is strongly dependent on baseline nutritional status: Syed et al. (2023) highlighted that most trials fail to account for pre-existing deficiencies, and that personalized supplementation based on biomarkers is essential for interpreting efficacy [89]. Similarly, Mauermann (2026) in a comprehensive JAMA review stressed that vitamin B12 deficiency is a treatable cause of neuropathy and that supplementation should be targeted to deficient patients rather than given indiscriminately [90]. The majority of our studies did not report baseline vitamin concentrations or did not stratify participants according to deficiency status. This is a critical limitation, as supplementation is likely to exert its greatest effect in individuals with pre-existing deficiency, while those with normal levels may derive little or no benefit. The lack of stratification by baseline vitamin status may therefore have diluted the overall treatment effect and contributed to the substantial heterogeneity observed across outcomes. Future trials should systematically measure relevant biomarkers (e.g., serum B12, methylmalonic acid, homocysteine, thiamine diphosphate) and restrict enrolment to patients with confirmed deficiency, or at least perform pre-specified subgroup analyses based on baseline levels. For example, Mansour et al. (2026) compared oral vitamin B12 at 1000 μg vs. 2000 μg daily in patients with DPN and low serum B12 (<200 pg/mL) and found that both doses similarly improved pain and MNSIE scores, with no added benefit from the higher dose [91]. This reinforces that even modest supplementation can be effective in deficient patients, and that dose escalation beyond a threshold may not yield additional neuropathic benefit.

### Limitations

Several limitations must be acknowledged:

1. For pain (I^2^ = 84.5%) and peroneal NCV (I^2^ = 87.6%), pooled estimates have high uncertainty and are presented narratively. Sources of heterogeneity could not be fully identified due to small numbers of studies.
2. Most outcomes were informed by only 4–6 studies, limiting statistical power for subgroup analyses and publication bias assessment.
3. Two studies combined B vitamins with other neuroactive agents (antioxidants, minerals, vitamins C/E), preventing specific attribution of effects to B vitamins.
4. A total of 11 of 13 studies did not measure baseline B-vitamin levels; no study stratified by deficiency status. This is the most critical gap for future research.
5. Pre-specified analyses by dose, duration, route, and baseline status could not be performed due to insufficient studies per subgroup.
6. Two studies had high risk of bias, and five had some concerns, potentially inflating effect estimates.
7. Embase, Web of Science, and Scopus were not searched due to institutional access limitations; however, the search of PubMed, Cochrane, and ClinicalTrials.gov, supplemented by reference screening of eight systematic reviews, likely captured the vast majority of published RCTs.
8. The significant result for the Pain Detect Questionnaire derives from two studies by the same investigators [69,71] and cannot be considered independently replicated evidence.
9. The statistically significant improvement in MNSIE does not meet the established MCID of 0.5 points; MCID thresholds for other outcomes are either absent or not met by the pooled estimates.

## 5. Conclusions

The findings of this meta-analysis must be interpreted in the context of substantial limitations: high heterogeneity for several outcomes, small numbers of studies per comparison, inclusion of multi-component interventions, and near-universal absence of baseline vitamin B status data. This meta-analysis provides evidence that vitamin B supplementation may improve certain clinical neuropathy scores (MNSIQ, MNSIE) and enhance sural nerve conduction velocity and amplitude in patients with diabetic peripheral neuropathy. However, no significant benefit was observed for pain intensity measured by generic scales (NRS/VAS) or for peroneal and tibial nerve parameters; also, the improvement in MNSIE (MD −0.39) did not meet the established MCID of 0.5 points. The results are characterized by substantial heterogeneity, largely driven by differences in study design (open-label vs. double-blind), patient populations (type 1 vs. type 2 diabetes), and intervention protocols (monotherapy vs. combination therapy). Notably, combination regimens containing multiple B vitamins showed more consistent benefits than single-agent supplementation, but formal subgroup analysis did not confirm statistically significant superiority over monotherapy. Given the conflicting findings and the high risk of bias in several included trials, routine prescription of vitamin B supplements for all DPN patients cannot be recommended at this time. However, in patients with documented B-vitamin deficiency (e.g., metformin-induced B12 deficiency) or those who fail to respond to first-line neuropathic pain agents, a trial of a standardized multi-B-vitamin combination may be justified. Future large, well-designed randomized controlled trials are urgently needed, with stratification by baseline vitamin status and consistent use of disease-specific outcome measures.

## Figures and Tables

**Table 1:** Baseline characteristics of included trials.

Study	Number	Country	Age (Intervention/Control)	Gender (Intervention/ Control)	DM Duration (Intervention/ Control)	Intervention	Control	Dose	Duration (Months)	Design	Co-Interventions	Baseline Vitamin Status	Primary Endpoints Assessed
Didangelos T., 2020 [62]	85	Greece	65.12 ± 11.16/62.34 ± 11.32	23 (53.5%)/21 (50%) male	15.01 ± 8.2/14.6 ± 9.7	SOD + ALA + ALC + B12	placebo	SOD 10 mg, ALA 570 mg, ALC 300 mg, B12 0.25 mg per day	12.0	prospective, double-blind, placebo-controlled	Yes: SOD, alpha-lipoic acid, and acetyl-L-carnitine are active non-B components; the independent contribution of B12 cannot be isolated	Baseline serum B12 reported in original article; no deficiency-stratified randomisation	Neuropathy symptoms/signs (MNSIQ/MNSIE), Pain Detect score, nerve conduction parameters
Purwata T. E., 2021 [63]	42	Indonesia	56.2 ± 7.0/59.5 ± 7.2	8 (38%)/9 (42%) male	4.2 ± 1.9/4.2 ± 1.4	methylcobalamin	placebo (saline) + oral amitriptyline 12.5 mg bid	500 μg intravenous on alternate days (total 5 injections over 9 days)	0.28	randomized, double-blind, placebo-controlled trial	Amitriptyline was given in both arms as background active analgesic therapy	Not reported	Pain intensity (NRS) and neuropathy symptom score/MNSIQ
Didangelos T., 2021 [64]	90	Greece	64.0 ± 7.8/61.7 ± 8.3	22 (50%)/26 (56.5%) male	14.0 ± 8.8/11.6 ± 5.9	methylcobalamin	placebo	1000 μg orally once daily	12.0	prospective, double-blind, placebo-controlled trial	None reported beyond usual diabetes care	Baseline serum B12 reported in original article; no deficiency-stratified randomisation	Neuropathy symptoms/signs (MNSIQ/MNSIE), pain outcomes, nerve conduction parameters
Fonseca V. A., 2013 [65]	214	USA	62.29 ± 8.54/62.95 ± 9.17	73 (68.9%)/75 (69.4%) male	11.4 ± 9.6/11.5 ± 8.6	L-methylfolate + methylcobalamin + pyridoxal-5′-phosphate	placebo	L-methylfolate 6 mg, methylcobalamin 4 mg, pyridoxal-5′-phosphate 70 mg per day	6.0	multicenter, randomized, double-blind, placebo-controlled trial	B-vitamin combination only; no non-B active co-intervention reported	Not reported	Neuropathy symptom scores (including MNSI/NTSS-related measures), pain, and nerve conduction parameters
Farvid M.S, 2011 [66]	44	Iran	54.4 ± 8.0/52.7 ± 8.5	13 (59.1%)/10 (45.5%) male	12.3 ± 7.6/8.6 ± 6.8	Zn, Mg, vit C, E, B1, B2, B6, biotin, B12, folic acid	placebo	Minerals (zinc 20 mg, magnesium 250 mg) + vitamins (C 200 mg, E 100 mg) + B vitamins (B1 10 mg, B2 10 mg, B6 10 mg, biotin 200 μg, B12 10 μg, folic acid 1 mg)—daily	4.0	randomized, double-blind, placebo-controlled clinical trial	Yes: zinc, magnesium, vitamins C and E, and biotin were co-administered; the B-vitamin-specific contribution cannot be isolated	Not reported	Neuropathy scores and nerve conduction parameters
Stracke H., 1996 [67]	24	Germany	58 (50–65)/60 (47–65)	6 (54.5%)/8 (61.5%) male	10 (4–25)/12 (1–40)	benfotiamine + pyridoxine + cyanocobalamin	placebo	Benfotiamine (40 mg) + pyridoxine HCl (vitamin B6, 90 mg) + cyanocobalamin (vitamin B12, 0.25 mg). Dosage: first 14 days: 2 capsules q.i.d.; then 1 capsule t.i.d.	3.0	randomized, double-blind, placebo-controlled study	B-vitamin combination only; no non-B active co-intervention reported	Not reported	Neuropathy symptoms and nerve conduction parameters
Stracke H., 2008 [68]	88	Germany	62 (47–73)/61 (42–72)	25 (53.2%)/24 (58.5%) male	13.2 (0.75–41)/11.7 (0.5–37)	benfotiamine	placebo	600 mg	1.5	double-blind, randomized, placebo-controlled phase-III trial	None reported	Not reported	Neuropathy symptom score/clinical neuropathy measures and pain-related outcomes
Ziegler D., 2026 [69]	51	Germany	n/a	n/a	n/a	benfotiamine	placebo	300 mg twice daily	12.0	randomized, double-blind, placebo-controlled parallel group phase-II trial	None reported	Not reported	Clinical neuropathy measures, nerve conduction parameters, and morphometric/neurophysiological outcomes
Kiew K. K., 2002 [70]	30	Malaysia	55.2 ± 7.0/54.5 ± 7.4	n/a	10.0 ± 3.7/7.0 ± 4.2	sulbutiamine	no treatment	400 mg/day orally	1.5	open randomized controlled study	None reported	Not reported	Neuropathy symptoms and peroneal nerve conduction/amplitude
Fraser D. A., 2012 [71]	59	Norway	n/a	n/a	DM type I, >15 years duration	benfotiamine	placebo	300 mg/day orally	24.0	parallel, randomized, double-blind, placebo-controlled prospective trial	None reported	Not reported	Peripheral nerve function/nerve conduction parameters and inflammatory markers
Stirban O.A., 2020 [72]	14	Germany	n/a	n/a	n/a	benfotiamine	placebo	600 (first 3 months), then 300	12.0	double-blind, randomized, placebo-controlled, parallel group pilot study	None reported	Not reported	Sensorimotor polyneuropathy clinical and neurophysiological measures
Levin E.R., 1981 [73]	18	USA	55.5 ± 3.6/56.7 ± 3.2	9/9 male (all male)	9.9 ± 5.2/13.7 ± 4.9	pyridoxine	placebo	50 mg t.i.d.	4.0	double-blind, randomized controlled trial	None reported	Not reported	Clinical neuropathy symptoms and nerve conduction parameters
Mottaghi T., 2019 [74]	75	Iran	54.9 ± 5.5/55.3 ± 6.0	25 (62.5%)/18 (45%) male	12.4 ± 3.2/11.9 ± 3.4	folic acid	placebo	1 mg/day orally	4.0	randomized, double-blind, placebo-controlled study	None reported	Not reported	Nerve conduction velocity parameters

Abbreviation: ALA, alpha-lipoic acid; ALC, acetyl-L-carnitine; B12, vitamin B12; bid, twice daily; DM, diabetes mellitus; q.i.d., four times daily; SOD, superoxide dismutase; t.i.d., three times daily. A Data presented as mean ± standard deviation; n/a = not available.

**Table 2:** Publication bias: Egger’s test results.

Outcome	Studies (k)	Egger’s Test, *p*-Value	Interpretation
MNSIE	5	0.553	No significant asymmetry
MNSIQ	4	0.618	No significant asymmetry (low power)
NRS Pain	4	0.571	No significant asymmetry (low power)
Peroneal NCV	6	0.360	No significant asymmetry
Peroneal Amplitude	4	0.849	No significant asymmetry (low power)
Sural NCV	5	0.388	No significant asymmetry
Sural Amplitude	5	0.304	No significant asymmetry

## Data Availability

The original contributions presented in this study are included in the article. Further inquiries can be directed to the corresponding authors.

## Supplementary Materials

The following supporting information can be downloaded at: https://www.mdpi.com/article/10.3390/jcm15135156/s1, Supplementary Material S1. Complete search strategy; Table S1. List of relevant systematic reviews.
