# Supplementary material for: Vitamin B Supplementation for Diabetic Peripheral Neuropathy: A Review and Meta-Analysis
Source: J Clin Med. 2026 Jul 2;15(13):5156. doi: 10.3390/jcm15135156 (PMC13362621; doi:10.3390/jcm15135156)
Supplement: Supplementary file 1 [file jcm-15-05156-s001.zip › jcm-4376984-supplementary.pdf]

Supplementary Material S1. Complete search strategy

1. *Database: PubMed*

Date search: March 14, 2026
Records retrieved: 585
Limits: No language restrictions. No filters.

Search string:
(diabetic AND neuropathy) AND ("vitamin B" OR "B vitamins" OR "thiamine" OR "vitamin B1" OR "benfotiamine" OR "vitamin B6" OR "pyridoxine" OR "vitamin B12" OR "cobalamin" OR "methylcobalamin" OR "folic acid" OR "folate" OR "vitamin B9")

2. *Database: Cochrane Database*

Date search: March 14, 2026
Records retrieved: 237
Limits: No language restrictions. No filters.

Search string:
(diabetic AND neuropathy) AND ("vitamin B" OR "B vitamins" OR "thiamine" OR "vitamin B1" OR "benfotiamine" OR "vitamin B6" OR "pyridoxine" OR "vitamin B12" OR "cobalamin" OR "methylcobalamin" OR "folic acid" OR "folate" OR "vitamin B9")

3. *Register: ClinicalTrials.gov*

Date search: March 14, 2026
Records retrieved: 48 trials
Limits: No language restrictions. No filters.

Search string:
(diabetic AND neuropathy) AND ("vitamin B" OR "B vitamins" OR "thiamine" OR "vitamin B1" OR "benfotiamine" OR "vitamin B6" OR "pyridoxine" OR "vitamin B12" OR "cobalamin" OR "methylcobalamin" OR "folic acid" OR "folate" OR "vitamin B9")

The literature search yielded 870 records in total: 822 from electronic databases (PubMed: 585, Cochrane: 237) and 48 from ClinicalTrials.gov.

Result: 870 articles and trial

---

4. *Database: Cochrane Database*

From the Cochrane Central Register of Controlled Trials, we retrieved 237 records matching our search strategy. After manual screening of titles, abstracts, and (when available) full texts, we excluded 104 records. The reasons for exclusion were categorised as follows: (1) not a randomised controlled trial (e.g., conference abstracts, reviews, case reports, pharmacoeconomic analyses) – 10 records; (2) wrong population (not diabetic peripheral neuropathy) – 16 records; (3) wrong intervention (B vitamins not studied, or their effect could not be separated from other active components) – 37 records; (4) complex intervention where the contribution of B vitamins could not be isolated – 15 records; (5) no adequate control group – 3 records; (6) no usable data for meta-analysis (e.g., trial registrations without published results, or missing numerical outcome data) – 22 records; and (7) duplicate publication – 1 record.The remaining records proceeded to full-text assessment and data extraction.

5. 108 duplicated were found between 133 left articles from Cochrane Database, 585 articles from PubMed and 48 trials from ClinicalTrials.gov.

Result: 658 articles and trial

---

6. The search was restricted to these three databases due to institutional subscription limitations precluding access to Embase, Web of Science, and Scopus. To partially compensate, reference lists of eight relevant systematic reviews were systematically screened (Table S1).

Table S1. List of relevant systematic reviews

References from:	Number of Research Items
Stein J, Geisel J, Obeid R. Association between neuropathy and B-vitamins: A systematic review and meta-analysis. *Eur J Neurol*. 2021;28(6):2054-2064. https://doi.org/10.1111/ene.14786	75
Huo J, Xue Y, Dong X, et al. Efficacy of vitamin and antioxidant supplements for treatment of diabetic peripheral neuropathy: systematic review and meta-analysis of randomized controlled trials. *Nutr Neurosci*. 2023;26(8):778-795. https://doi.org/10.1080/1028415X.2022.2090606	47
Ang CD, Alviar MJ, Dans AL, et al. Vitamin B for treating peripheral neuropathy. *Cochrane Database Syst Rev*. 2008;2008(3):CD004573. https://doi.org/10.1002/14651858.CD004573.pub3	34
Karedath J, Batool S, Arshad A, et al. The Impact of Vitamin B12 Supplementation on Clinical Outcomes in Patients With Diabetic Neuropathy: A Meta-Analysis of Randomized Controlled Trials. Cureus. 2022;14(11):e31783. https://doi.org/10.7759/cureus.31783	25
Farah S, Yammine K. A systematic review on the efficacy of vitamin B supplementation on diabetic peripheral neuropathy. *Nutr Rev*. 2022;80(5):1340-1355. https://doi.org/10.1093/nutrit/nuab116	41
Khalil H, Ang CD, Khalil V. Vitamin B for treating diabetic peripheral neuropathy - A systematic review. *Diabetes Metab Syndr*. 2021;15(5):102213. https://doi.org/10.1016/j.dsx.2021.102213	75
Muhamad R, Akrivaki A, Papagiannopoulou G, Zavridis P, Zis P. The Role of Vitamin B6 in Peripheral Neuropathy: A Systematic Review. Nutrients. 2023;15(13):2823. https://doi.org/10.3390/nu15132823	32
Sawangjit R, Thongphui S, Chaichompu W, Phumart P. Efficacy and Safety of Mecobalamin on Peripheral Neuropathy: A Systematic Review and Meta-Analysis of Randomized Controlled Trials. *The Journal of Alternative and Complementary*	61

Medicine: Paradigm, Practice, and Policy Advancing Integrative Health. 2020;26(12):1117-1129. https://doi.org/10.1089/acm.2020.0068	

Result: 390 articles

Of the 390 records initially identified from reference lists, 77 were classified as clearly off-topic during preliminary review. Therefore, 313 candidate records were retained. After removing duplicates within this set (n = 53), 260 unique review-derived records remained.

7. The database records (n = 658) and review-derived records (n = 260) were combined, yielding 918 records. After removing duplicates between these two sources (n = 56), a total of 862 unique records were screened at the title and abstract level.

8. During title/abstract screening, 839 records were excluded as they did not meet for the following reasons: (1) not a randomised controlled trial (e.g., conference abstracts, reviews, case reports, pharmacoeconomic analyses) – 220 records; (2) wrong population (not diabetic peripheral neuropathy) – 106 records; (3) wrong intervention (B vitamins not studied, or their effect could not be separated from other active components) – 189 records; (4) complex intervention where the contribution of B vitamins could not be isolated – 75 records; (5) no adequate control group – 44 records; (6) no usable data for meta-analysis (e.g., trial registrations without published results, or missing numerical outcome data) – 161 records; (7) duplicate publication – 19 records; and (8) animal or laboratory studies – 25 records.

9. The remaining 23 reports were sought for full-text retrieval. Three full texts could not be obtained, leaving 20 reports for detailed eligibility assessment.

10. After full-text evaluation, 7 reports were excluded for the following reasons: inadequate control group (n = 2), insufficient data for meta-analysis - datasets without statistics (n = 4) and same-cohort analysis - duplicate (n=1).

11. 13 randomized controlled trials met all inclusion criteria and were included in the systematic review.
